# Co-expression network analysis identified hub genes critical to triglyceride and free fatty acid metabolism as key regulators of age-related vascular dysfunction in mice

**DOI:** 10.18632/aging.102275

**Published:** 2019-09-12

**Authors:** Huimin Li, Xinhui Wang, Xinyue Lu, Hongxin Zhu, Sheng Li, Shiwei Duan, Xinzhi Zhao, Fuquan Zhang, Gil Alterovitz, Fudi Wang, Qiang Li, Xiao-Li Tian, Mingqing Xu

**Affiliations:** 1 Bio-X Institutes, Key Laboratory for the Genetics of Developmental and Neuropsychiatric Disorders (Ministry of Education), Shanghai Jiao Tong University, Shanghai 200030, China; 2 Center for Biomedical Informatics, Harvard Medical School, Boston, MA 02115, USA; 3 School of Public Health, The First Affiliated Hospital, Institute of Translational Medicine, Zhejiang University School of Medicine, Hangzhou 310058, China; 4 Medical Genetics Center, School of Medicine, Ningbo University, Ningbo 315000, China; 5 International Peace Maternity and Child Health Hospital of China Affiliated to Shanghai Jiao Tong University, Shanghai 200030, China; 6 Wuxi Mental Health Center, Wuxi 214000, China; 7 Translational Medical Center for Development and Disease, Institute of Pediatrics, Shanghai Key Laboratory of Birth Defect, Children's Hospital of Fudan University, Shanghai 201102, China; 8 Department of Human Population Genetics, Human Aging Research Institute and School of Life Science, Nanchang University, Nanchang 330031, China

**Keywords:** co-expression network, hub gene, module, aging, vascular dysfunction, mouse

## Abstract

Background: Aging has often been linked to age-related vascular disorders. The elucidation of the putative genes and pathways underlying vascular aging likely provides useful insights into vascular diseases at advanced ages. Transcriptional regulatory network analysis is the key to describing genetic interactions between molecular regulators and their target gene transcriptionally changed during vascular aging.

Results: A total of 469 differentially expressed genes were parsed into 6 modules. Among the incorporated sample traits, the most significant module related to vascular aging was associated with triglyceride and enriched with biological terms like proteolysis, blood circulation, and circulatory system process. The module associated with triglyceride was preserved in an independent microarray dataset, indicating the robustness of the identified vascular aging-related subnetwork. Additionally, Enpp5, Fez1, Kif1a, F3, H2-Q7, and their interacting miRNAs mmu-miR-449a, mmu-miR-449c, mmu-miR-34c, mmu-miR-34b-5p, mmu-miR-15a, and mmu-let-7, exhibited the most connectivity with external lipid-related traits. Transcriptional alterations of the hub genes Enpp5, Fez1, Kif1a, and F3, and the interacting microRNAs mmu-miR-34c, mmu-miR-34b-5p, mmu-let-7, mmu-miR-449a, and mmu-miR-449c were confirmed.

Conclusion: Our findings demonstrate that triglyceride and free fatty acid-related genes are key regulators of age-related vascular dysfunction in mice and show that the hub genes for Enpp5, Fez1, Kif1a, and F3 as well as their interacting miRNAs mmu-miR-34c, mmu-miR-34b-5p, mmu-let-7, mmu-miR-449a, and mmu-miR-449c, could serve as potential biomarkers in vascular aging.

Methods: The microarray gene expression profiles of aorta samples from 6-month old mice (n=6) and 20-month old mice (n=6) were processed to identify nominal differentially expressed genes. These nominal differentially expressed genes were subjected to a weighted gene co-expression network analysis. A network-driven integrative analysis with microRNAs and transcription factors was performed to define significant modules and underlying regulatory pathways associated with vascular aging, and module preservation test was conducted to validate the age-related modules based on an independent microarray gene expression dataset in mice aorta samples including three 32-week old wild-type mice (around 6-month old) and three 78-week old wild-type mice (around 20-month old). Gene ontology and protein-protein interaction analyses were conducted to determine the hub genes as potential biomarkers in the progress of vascular aging. The hub genes were further validated with quantitative real-time polymerase chain reaction in aorta samples from 20 young (6-month old) mice and 20 old (20-month old) mice.

## INTRODUCTION

Vascular aging is characterized by structural and functional alterations of the vascular wall, deteriorating vascular integrity and vessel homeostasis, including increased luminal diameter with wall remodeling and augmented intimal and medial thickening, reorganization of the extracellular matrix with altered collagen, and elastin content and calcifications [1]. These changes may increase cardiovascular morbidity and mortality by leading unequivocally to a number of detrimental changes in the cardiovascular system that are discriminated with atherosclerotic pathology [2]. These age-related vascular diseases remain a leading cause of mortality and account for 40% of overall deaths worldwide [3]. Aging-induced aorta stiffening was demonstrated to be a natural consequence of increasing age that might be due to the several impairments including alteration in aortic wall [4]. The development of physiological vascular aging is subjected to the alterations in the function of the endothelium that line the lumen of blood vessels, which are mediated through both genetic and environmental factors [5–7]. At the cellular level, decreased protein synthesis, increased angiotensin II levels, mitochondrial dysfunction, autophagy, altered pattern of calcium regulation and increased DNA, protein, and lipid oxidation are mainly observed [8–10]. Additionally, it is evident that processes involving immune responses and oxidative stress occur in vascular aging [11].

The elucidation of the putative genes and pathways underlying vascular aging is critical for understanding the molecular mechanisms of age-related vascular diseases. Previous research has discussed the roles of Fat1 in controlling mitochondrial function and cell growth [12], transcriptional regulator HHEX [13], and FOXOs/sirtuins angiogenesis [14, 15] in vascular aging and conducted extensive research to understand heart diseases including vascular dysfunction. With the development of high-throughput microarray technology, gene expression profiles have been used to identify genes and pathways associated with the pathogenesis of vascular aging, which has helped to partially illustrate the underlying mechanisms. For example, two transcriptomic studies identified some differentially expressed genes related to age-related vascular changes based on gene expression profiling in mice [16, 17]. However, these studies put emphasis only on screening differentially expressed genes (DEGs) rather than determining the connection between them, in which genes with similar expression patterns may be functionally related. Moreover, the regulatory interactions between genes in particular pathways or biological processes across multiple vascular aging stages have not been investigated. Additionally, potential novel regulators of transcription and post-transcription of age-related vascular gene expression, including micro-RNAs, long noncoding RNAs, and transcription factors, have not been investigated how they regulate the transcription-level mRNA interactions. Gene interactions in vascular aging lead to endothelial cell metabolism, thereby understanding the functional molecular mechanisms regulated by these interactions is essential for gaining biological insights into cellular functions, predicting downstream events, and ideally manipulating the aging process based on desired goals. Co-expression network analysis enables us to cluster genes by assigning them to known biological functions in which they are involved [18]. Among the co-expression network inference algorithms, weighted gene co-expression network analysis (WGCNA) is a relatively new statistical method not only to infer correlation pattern between two genes but also covers neighborhood across expression data [19] while constructed networks generally could be divided to modules. Plenty of evidence suggested the modules as stable units underlying transcriptional regulation networks whose function can remain the same while individual gene expression can be changed or replaced by other genes with similar redundant functions [20].

In this study, we conducted a co-expression network analysis for identifying putative genes and pathways involved in a triangle of aging, vascular dysfunction, and lipid levels. A network-driven integrative analysis was performed to find significant modules and module preservation test was conducted to test the robustness of the significant modules. Further gene ontology and protein-protein interaction analyses were conducted to determine potential biomarkers in the progress of vascular aging with wet-lab verifications. Through co-expression network analysis adopted by WGCNA, we identified triglyceride-related traits correlated with gene expression changes in mouse aorta controlled by aging. Functional analysis of the top modules implies a relationship between aging-related transcriptional changes and fat components in aorta.

## RESULTS

### Network construction and module detection

**Figure 1:**
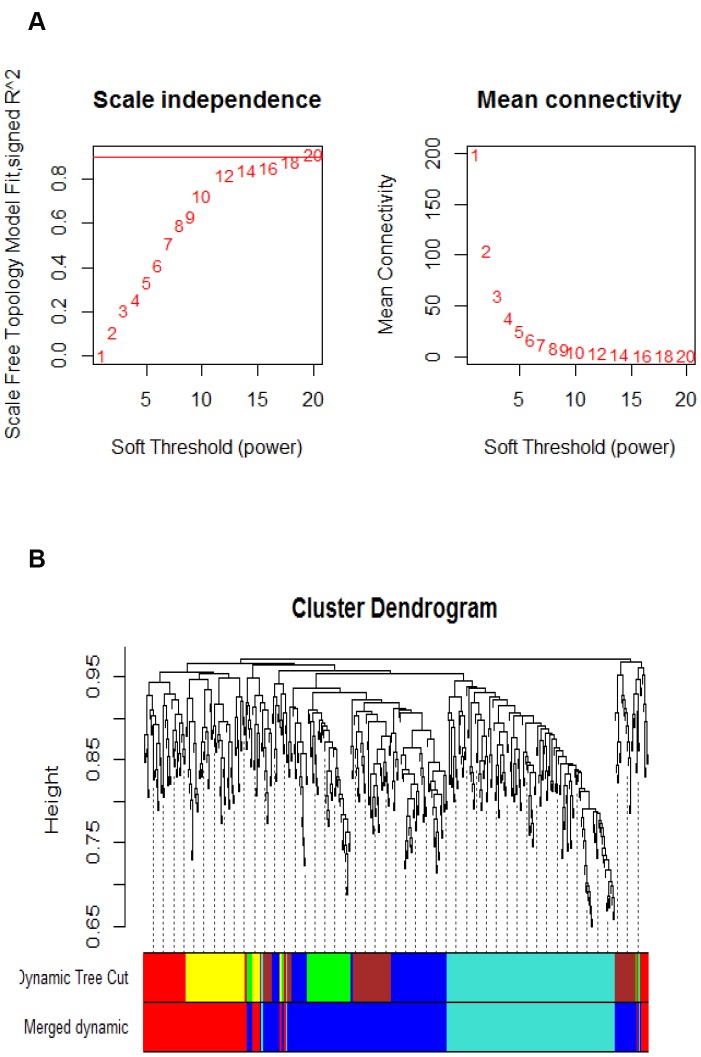
**Parameter analysis of inferred co-expression network and modules.** (**A**) A scaling factor beta determination based on the scale-free topology criterion. (**B**) Hierarchical clustering of genes in significant modules. The colors are assigned to each module by the Dynamic Tree Cut algorithm.

After microarray data preprocessing, a total of 2,256 differentially expressed Agilent transcripts were left. We further removed ambiguous probes and duplicated genes. The ambiguous probes included positive and negative controls in the microarray, and the duplicated genes were removed according to their gene symbols. Finally, the expression values of the 469 differential genes were selected for subsequent analysis. In graph theory, the nodes with higher connections are more likely to be pivotal connectors. Likewise, critical points controlling important dynamic components in biological networks [21], removal of them may cause biological systems failure in saving their coherence. In order to identify such interconnected genes from aging-associated co-expression network abstractly, adjacency matrix was obtained from the Pearson correlation matrix with a power β=12 based on the scale-free topology criterion [21]. Subsequently, the represented matrix was transformed to similarity matrix in clustering process during which genes grouped in a cluster were likely to be biologically relevant to the same pathway. The value of power β=12 could emphasize robust correlations and remove unreliable correlations between genes on an exponential level. Figure 1A shows the determination of β parameter based on the description in the WGCNA manual. Briefly, given Agilent series GSE50833, we used the WGCNA to establish a number of modules in co-expression network, and 6 modules were obtained (Figure 1B). As illustrated in Figure 1B, modules in gene dendrogram are shown in different colors and based on dynamic branch cutting algorithm underneath row color assigns the modules membership.

### Modules associated with vascular aging and lipid levels

**Figure 2:**
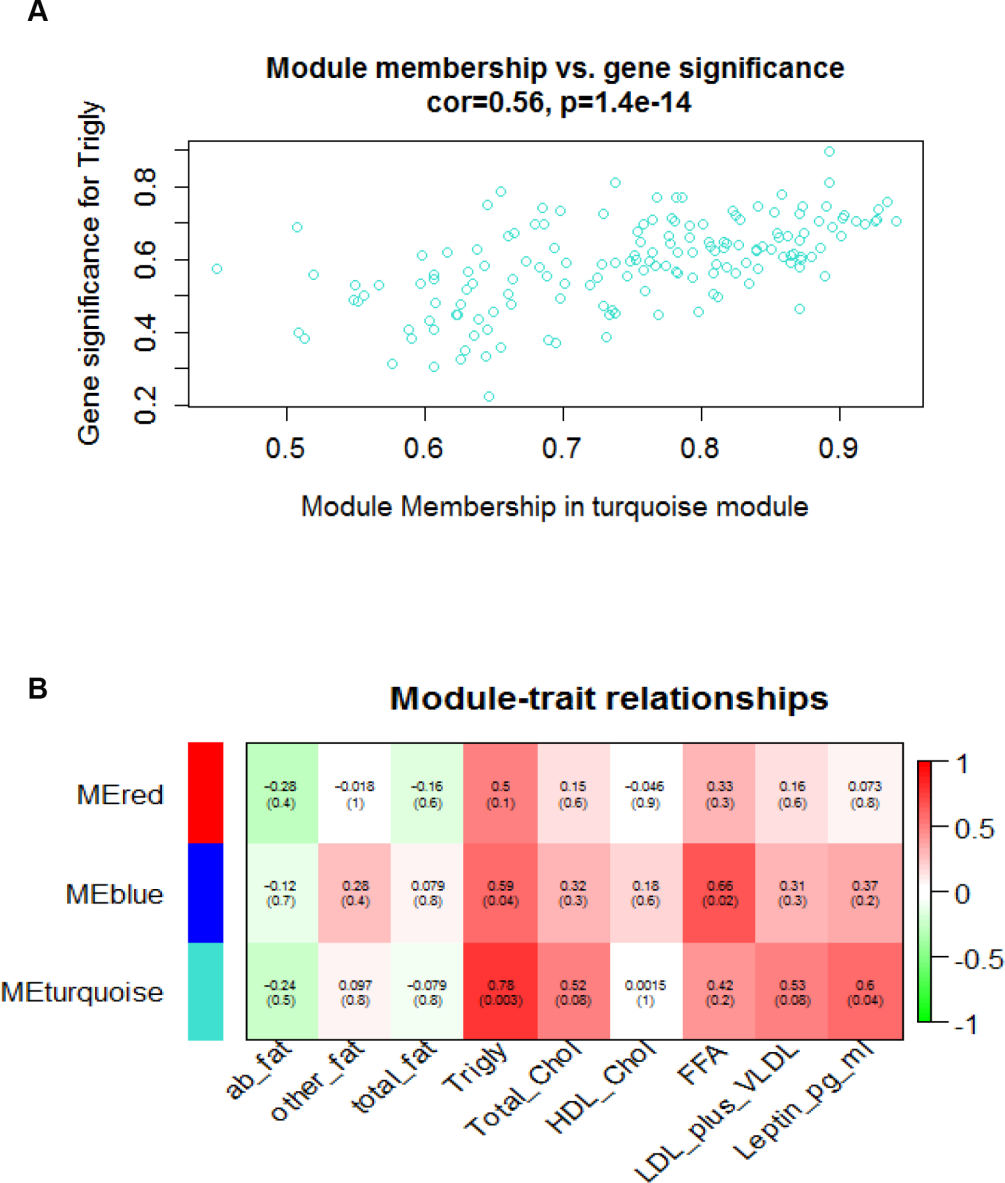
**Module-traits and module_membership-gene_significance correlation analyses.** (**A**) Scatterplot shows a highly significant correlation between gene significant (GS) versus module membership (MM) in the turquoise module with Triglyceride. (**B**) Heatmap shows correlation between assayed traits and module eigengene values. Green and red colors represent the negative and positive correlation respectively. Decimals outside of round brackets are correlation, and decimals inside of round brackets stand for gene significance level.

We aimed to gain new insights into the roles of specific modules that might be involved in vascular aging, module eigengenes associated with phenotypic traits were assessed in this study. An eigengene is referred to as gene expression profiles inside each of the modules summarized by the first principle component. The turquoise and blue modules that are positively correlated with triglyceride, leptin, and free fatty acid (FFA) respectively, were established to be significantly associated to vascular aging stage with an absolute correlation coefficient of > 0.6 and p-value of < 0.05. Figure 2A shows a highly significant correlation between gene significant (GS) versus module membership (MM) in the turquoise module with Triglyceride, and Figure 2B illustrates the correlation between turquoise module size and triglyceride. Next, we examined whether significant modules (turquoise and blue) were enriched for GO terms relevant to age-related vascular disorders. Gene ontology enrichment analysis for the turquoise module is presented in Table 1 and the GO analysis of other modules are presented in Supplementary Table 5. From Table 1, we noticed that 44 genes are involved in the proteolysis biological process, and the products of 46 genes are located under membrane-bounded vesicle.

**Table 1:** GO enrichment analysis of genes assigned to the turquoise module.

**Category**	**Term**	**Counts of genes**	***P-value***
CC	extracellular matrix	16	0.000855
CC	mitochondrial envelope	10	0.0017607
CC	mitochondrial membrane	10	0.0017607
CC	neuron projection	23	0.0048588
BP	Proteolysis	24	0.0061265
CC	neuron part	26	0.0064492
BP	Circulatory system process	14	0.0065327
BP	Blood circulation	14	0.0065327
CC	Membrane-bounded vesicle	45	0.0072194
CC	Organelle inner membrane	6	0.0074282

BP: biological process; CC: cellular component

### Inference of miRNA–mRNA-transcription factor interaction

**Figure 3:**
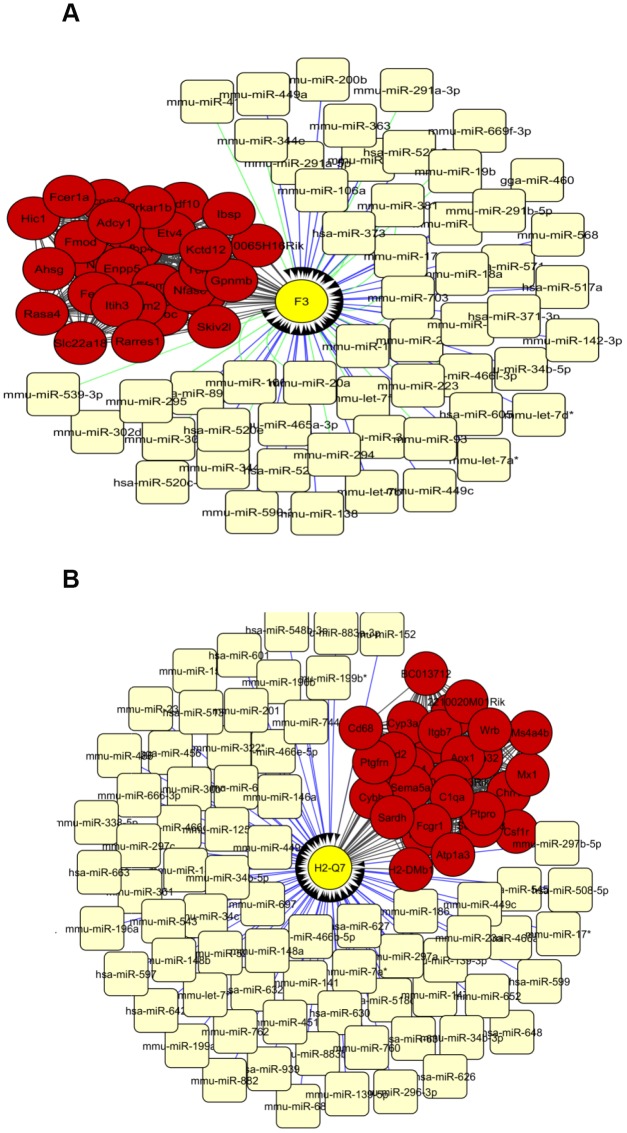
The network of top 30 interconnected genes in the turquoise (**A**) and blue (**B**) modules and predicted miRNAs from TargetScan and Microcosm. The hub genes are shown in red and miRNAs in light yellow. mRNA-miRNA interactions from TargetScan and Microcosm are shown in green and blue lines respectively. Yellow circles show the genes that are putatively regulated by miRNAs.

The top 30 interconnected genes within each of the turquoise, blue and red modules were graphically depicted by utilizing Cytoscape (Supplementary Table 17, Supplementary Table 19, Supplementary Table 21). Interestingly, miRNA-target analysis showed that in the turquoise module, gene F3 is putatively targeted by 23 and 38 predicted miRNAs from TargetScan and MicroCosm respectively (Figure 3A). Additionally, in the blue module, gene H2-Q7 illustrated to be targeted by 78 miRNAs in MicroCosm (Figure 3B). No genes were found to be targeted with miRNAs in the red module. Among the miRNAs, mmu-miR-449a, mmu-miR-449c, mmu-miR-34c, mmu-miR-34b-5p, mmu-miR-15a, and mmu-let-7 were listed as common between networks built by turquoise and blue modules. Due to the inconvenience of considering a highly dimensional set of transcription factors, we only investigated transcription factors in a network of the top 90 connected hub nodes depicted for mRNA-miRNA interaction analysis (Supplementary Table 9), when transcription factors Pax8 and Hsf1 were ranked as the most significant based on the normalized enrichment score test (NES) (Table 2).

**Table 2:** Promoter analysis of the top 90 hub genes obtained from three modules significant associated with vascular aging status.

**Transcription factor**	**NES**	**No. of targets**	**No. o Motifs/Tracks**
Pax8	4.484	25	7
Hsf1	4.04	11	1
Pou5f1	3.916	27	2
Ovol1	3.78	14	3
Lef1	3.589	12	4
Zic2	3.485	17	2
Rfx2	3.464	6	3
Egr1	3.433	14	3
Gata2	3.422	12	1
Gltpd1	3.287	11	2
Hic1	3.224	11	3
Zfat	3.212	22	3
Sod1	3.14	4	1
Irf4	3.047	10	1
Mtf1	3.034	11	2

NES: normalized enrichment score

### Module preservation test

To test if the age-dependent modules are preserved between the constructed network and a reference network, we built a reference network based on 1036 significantly differentially expressed transcripts with *P* < 0.05 from 6-month old wild-type mice (n=3) and 20-month old wild-type mice (n=3) in the GSE10000 dataset (Supplementary Table 3). Among the 2,256 transcripts from GSE50833 datasets, 125 genes (according to the gene symbols) were matched with modules defined by Ghazalpour et al. [22] (Supplementary Table 4), by which we had already built a reference network. Median rank and Z-summary values were calculated to conduct module preservation test, and both measures revealed high preservation of green and blue modules between the two networks (Supplementary Figure 5A and 5B). Largely overlapped interactions between the two networks were found in this study, indicating the robustness of the built modules is strong. The strong module preservation could be due to the overlapped interactions between the two networks, which indicate that the gene interactions within a module are conserved.

### qPCR validation of the key genes

To verify the main conclusion drawn from the microarray results, the relative expression levels of the five key genes (Enpp5, Fez1, Kif1a, F3, and H2-Q7) and their interacting microRNAs (mmu-miR-449a, mmu-miR-449c, mmu-miR-34c, mmu-miR-34b-5p, mmu-miR-15a, and mmu-let-7) were determined using qPCR. No significant difference in body weight was detected between the two groups of male mice. The RT-qPCR analysis results indicated that Enpp5, Fez1, Kif1a, F3, mmu-miR-34c, mmu-miR-34b-5p, and mmu-let-7 were significantly up-regulated in samples from 20-month old mice group compared with the 6-month old mice group, whereas mmu-miR-449a and mmu-miR-449c were significantly down-regulated. Meanwhile, there were no significant differences observed in the H2-Q7 and mmu-miR-15a genes between the two groups, but the expression level of H2-Q7 is a little higher in the group of old mice compared to the young mice.

## DISCUSSION

The purpose of this investigation was to identify fundamental mechanisms leading to transcriptional changes in mouse aorta toward aging with a network biology approach. Given the most variable transcripts in aorta between 6-month old mice (n=6) and 20-month old mice (n=6) as a discovery dataset, we built a weighted co-expression network. This network-based approach summarizes genes with similar expression profiles into the same modules, and therefore, co-grouped genes are thought to be involved in the same regulatory pathways [23]. When employing the Dynamic Tree Cut method, we detected 6 modules ranging from 160 genes in the turquoise module to 50 genes in the red and green modules. After merging modules with highly correlated eigengenes, the turquoise, blue and red modules were chosen for subsequently functional analysis (Supplementary Figures 1–4). To reveal vascular transcriptome’s relationship to age, we further detected trait-related gene modules and hub genes by incorporating lipid-related traits, such as triglycerides and free fatty acid, into the network analysis. Modules that are closely associated with clinical traits, might be biologically important in bridging aging and the incidence of vascular disorders. Therefore, it could draw an interaction between these traits and variable aging-associated gene expression changes in mouse aorta. The red module showed no correlation with assayed traits while the most significant and correlated module with the incorporated traits was turquoise (Figure 2, Supplementary Figure 7 and Supplementary Table 11).

Functional enrichment analysis of this module with DAVID software identified several terms apparently associated with aging-related vascular dysfunction, including extracellular matrix, blood circulation, circulatory system process, and proteolysis. Ponticos M et al. reported the cross-talk between extracellular matrix and vascular disorders in detail [24]. Blood vessels are distended by blood pressure and, therefore, require ECM components with elasticity yet with enough tensile strength to resist rupture and stiffness.

Our clinical implication of significant modules indicated that triglyceride and leptin levels are correlated with the turquoise module, and triglyceride and free fatty acid (FFA) levels are correlated with blue module, respectively. Whereas the correlation between triglyceride level with turquoise module was higher than that with blue module (Supplementary Figure 6 and Supplementary Table 11). Noblet et al. suggested that leptin is a key modulator in pathways involved in vascular proliferation [25]. Additionally, leptin sensitivity pointed to be declined during aging in rodents [26]. People found that hypertriglyceridemia may act as a prevalent risk factor for cardiovascular disease [27] and free fatty acid level may increase with aging [28].

Through module preservation analysis, our findings were strongly validated in the blue and green modules in an independent reference network, and the blue module was more preserved between the two networks and enriched with genes involved in immune response, leukocyte differentiation and hemopoiesis. In concordance, the study from Stervbo U et al. [29]. demonstrated that age-dependent detrimental alterations in leukocytes may dysfunction the innate immune system. Additionally, Babio N et al. [30] disclosed an association between leukocyte counts with hypertriglyceridemia. The free fatty acid may activate leukocytes following endothelial dysfunction through enhanced angiotensin II production [31].

The WGCNA package also computed two values as gene significance (GS) and module membership (MM) when the smaller corresponding p-value denotes higher Pearson’s correlation coefficient between gene expression profiles, incorporated clinical traits and modules, respectively (2) and candidate mRNAs with the highest GS and MM could be considered as the one significantly associated with phenotypic trait and module eigengene of a given module (Supplementary Table 10, Supplementary Table 11,  Supplementary Table 12). Hereby we focused on the turquoise module, the most correlated with triglyceride level. The top 13 genes with higher MM and GS were selected as the most biologically correlated with triglyceride level and turquoise module respectively (Table 3). Consequently, Fez1, Kifla, and Enpp5 were interesting genes as they were common among the top 30 connected genes visualized by Cytoscape (Figure 2B) and highly ranked genes based on GS and MM scores. Fez1 encodes an elongation protein that abnormally aggregated in aged mice [32]. Kif1a was exhibited as a crucial regulator of synaptic aging [33]. These highly connected inter-modular genes can be considered hubs and likely to play pivotal roles in maintaining the network functions.

**Table 3:** List of genes most correlated with triglyceride and turquoise module eigengene.

**Gene**	**P-value (GS)**	**Gene**	**P-value (MM)**
*Enpp5*	7.51E-05	*Igfbp4*	5.11E-06
*Fez1*	0.0013665	*Kif1a*	8.62E-06
*Il3ra*	0.0013893	*Rasa4*	1.32E-05
*Slc6a15*	0.0024338	*Skiv2l*	1.44E-05
*Igfbp2*	0.0029863	*Myoc*	1.51E-05
*Ache*	0.0032684	*Nfasc*	2.42E-05
*Spry1*	0.003308	*Kctd12*	3.55E-05
*Crlf1*	0.0033796	*Slc22a18*	5.59E-05
*Kif1a*	0.0042745	*Ibsp*	5.98E-05
*Ier3*	0.0049655	*Hic1*	6.21E-05
*Etv4*	0.0054145	*Prkar1b*	8.48E-05
*F3*	0.005536	*Fez1*	9.26E-05
*Mbd2*	0.005852	*Enpp5*	9.31E-05

GS: gene significance, MM: module membership

Centrality analysis of genes within turquoise and blue modules (Supplementary Table 13, Supplementary Table 14) by use of CytoNCA [34] revealed that Enpp5 had the highest between-centrality among the first 30 genes (Supplementary Table 15). Enpp5 is known to have catalytic activity, and its expression is changed in adipocytes transfected with Agt-shRNA, indicating that this gene is involved in blood pressure and lipid accumulation [35]. String protein-protein interaction networks of Enpp5 was presented in Supplementary Figure 8. It was also shown that Enpp5 acts as an extracellular signaling molecule in a broad variety of tissues [36]. In a word, lipid components, including triglyceride and free fatty acid, may inhibit the activities of blood cells to cause plying in immune system fallowing by vascular endothelial disruption. According to the GO analysis, genes F3, H2-Q7 and Enpp5 are therefore considered to be key genetic elements in age-related vascular changes. F3 is related to angiogenesis and coagulation cascades and was suggested to be involved in pathways that are relevant to apoptosis in aging mice [37]. H2-Q7 is a murine nonclassical MHC class I gene involved in the modulation of immune responses, whose expression showed gradual increase with aging [38]. The differential expression of H2-Q7 may contribute to the distinct patterns of mouse susceptibility/resistance to infectious and noninfectious disorders. In a recent study [39], miRNA families mir-34, mir-15 and mir-449 exhibited significant distinct expression patterns along with aging in mice. The levels of miR-34c are elevated in the hippocampus of AD patients and corresponding mouse models [40]. Guo et al. demonstrated kallistatin may reduce vascular aging by regulating microRNA-34a-SIRT1 pathway [41]. miRNA let-7 plays a role in tissue homeostasis, repair, and stem cell aging [42].

The key genes identified through our co-expression network are concordant with other aging-related findings. The availability of massive transcriptomic data has facilitated the reconstruction of biological networks, through which we are able to decipher how genes are interacted within intricate networks underlying age-related vascular disorders. We noted that, in agreement with previous significant experimental confirmation, network mining is robust to identify hub genes and depict the structural and functional features of biological networks. Compared to methods solely based on single genes derived by differential expression analysis, biological modules may represent more credible information. In frame of accurate approaches of network reconstruction and modularity analysis, we identified the hub genes Enpp5, Fez1, Kif1a, F3, H2-Q7, two transcription factors Pax8 and Hfs1, and their interacting miRNAs mmu-miR-449a, mmu-miR-449c, mmu-miR-34c, mmu-miR-34b-5p, mmu-miR-15a, and mmu-let-7, whose interactions could lead to age-related vascular dysfunctions.

Transcriptional alterations of the hub genes Enpp5, Fez1, Kif1a, and F3, and the interacting microRNAs mmu-miR-34c, mmu-miR-34b-5p, mmu-let-7, mmu-miR-449a, and mmu-miR-449c between 20 pairs of 6-month and 20-month old mice were confirmed by RT-PCR in our separate investigation. Whereas, no significant differences were observed in the H2-Q7 and mmu-miR-15a genes between the two groups, even though the expression level of H2-Q7 is a little higher in the group of old mice compared to the young mice, which may be due to the limited sample size.

We still need to be cautious to understand the biological network related to vascular aging in mice. First, the small sample size limited the findings and the results would be more reliable when utilizing a more adequate number of samples. Second, network analysis at transcriptome level could be more intensified through merging studies with entire protein-protein network to draw more precise conclusions regarding predicted hub genes and master regulators. Finally, we inferred an undirected network in which connectivity between nodes does not indicate the causal regulatory relationships.

In conclusion, triglyceride, free fatty acid, and leptin were significantly and positively correlated with age-related transcriptional changes in mice aorta. Meanwhile, five hub genes Enpp5, Fez1, Kif1a, F3, H2-Q7, two transcription factors Pax8 and Hfs1, and their interacting miRNAs mmu-miR-449a, mmu-miR-449c, mmu-miR-34c, mmu-miR-34b-5p, mmu-miR-15a, and mmu-let-7 exhibited the most connectivity with external lipid-related traits, and their interactions could lead to age-related vascular dysfunctions. Transcriptional alterations were confirmed with RT-PCR for the hub genes Enpp5, Fez1, Kif1a, and F3, and the interacting microRNAs mmu-miR-34c, mmu-miR-34b-5p, mmu-let-7, mmu-miR-449a, and mmu-miR-449c, which could serve as potential biomarkers in vascular aging.

## MATERIALS AND METHODS

### Data collection

We carefully selected vascular aging-associated dataset from the National Center for Biotechnology Information (NCBI) Gene Expression Omnibus (GEO, http://www.ncbi.nlm.nih.gov/geo/) database, and identified two time-series microarray datasets regarding vascular aging in mice aorta (GEO access number: GSE50833; GSE10000). The Agilent TXT files for GSE50833 and Affymetrix TXT files for GSE10000 were downloaded from the GEO database respectively. The data GSE50833 [16] was used as first-stage discovery set for co-expression network construction, and GSE10000 [17] as a second-stage independent verification dataset for module preservation test. This dataset GSE50833 consists of a total of 12 samples with data generation platform of GPL10787, and these samples correspond to 6-month old mice (n=6) and 20-month old mice (n=6). The data GSE10000 consists of a total of 18 samples with data generation platform of GPL1261 and GPL8321 respectively, and these samples correspond to three pairs of 6-week old wild-type and ApoE-/- mice, three pairs of 32-week old wild-type and ApoE-/- mice, and three pairs of 78-week old wild-type and ApoE-/- mice.

### Data preprocessing and identification of DEGs

Statistical software R (version 3.5.1) and its related packages were used for data preprocessing and identifying nominal DEGs between the two aging stages. The affyPLM package could fit the original data of the microarray to generate the weights and residuals diagram, the relative log expression (RLE), and the relative standard deviation (NUSE, Normalized unscaled standard errors) box diagram. Before we analyzed the data, we conducted quality testing of microarray data. In this process, we used some powerful and accurate R packages, such as affyPLM, affy, and RColorBrewer. After conducting the procedure of removing unqualified samples or probes, we got a reasonable and useful sample set. Raw files were normalized with quantile method (Supplementary Table 1) and the limma package (Linear Model for Microarray Data) was applied to extract genes with *P* < 0.05 by comparing the gene expression values between the 6- and 20-month old mice. These genes were considered as nominal DEGs without multiple test correction. Ultimately, the extracted Agilent probe IDs of the identified DEGs for the data GSE50833 were transformed into Agilent MIT IDs (Supplementary Table 2) and used for follow-up analysis.

### Network construction

After removing the redundant probes that were not able to contribute greatly to the construction of modules, the nominal DEGs were used to build weighted gene co-expression network through the WGCNA R package, by which the calculated absolute value of the Pearson correlation coefficient for all pair-wise comparisons of gene expression values was transformed into a similarity matrix. Next, the similarity matrix was applied to stepBystep, a network construction, and module detection function. We carried out this step by building a weighted matrix with a scaling factor-beta based on the assumption that biological networks are scale-free [43]. The modules were computed by assigning a minimum of 30 genes per module and keeping the default value of SplitDepth in two for a medium sensitivity of cluster splitting. These parameters would optimize scale-free topology and robust node connectivity criteria when any two genes were connected, and the edge weight was determined by the topological overlap matrix (TOM). Furthermore, genes were clustered into modules by utilizing average linkage hierarchical clustering using topological overlap dissimilarity matrix (1-TOM) as the distance measure, and modules were determined by the dynamic hybrid tree cut algorithm. Finally, similar modules whose eigengenes were highly correlated were merged to trim genes whose correlation with module eigengene was less than the defined threshold (hereby we used 0.25 as a threshold for merging similar modules). WGCNA determines highly inter-connected nodes as modules designated with different colors. Moreover, this algorithm is able to relate the identified modules to external clinical outcomes, and to export the network information to external software for visualization, e.g. VisANT (http://visant.bu.edu/) [44] or Cytoscape [45] that visualizes biological interactions between subnetworks.

### Gene ontology analysis and visualization

Gene Ontology (GO) analysis was conducted to reveal biological functions of gene products within significant modules from three scenarios: biological process (BP), cellular component (CC), and molecular function (MF) by using the software named Database for Annotation, Visualization, and Integrated Discovery (DAVID) [46]. DAVID facilitates high throughput gene functional analysis by accepting the input of a list of genes or an individual gene. Potential enriched functions of the DEGs in the determined modules were analyzed by the DAVID tool.

The top 30 high-rank intramedullary hub genes were prepared for visualization with VisANT (Supplementary Table 16, Supplementary Table 17, Supplementary Table 18, Supplementary Table 19, Supplementary Table 20, Supplementary Table 21).

### Modeling miRNA–mRNA-transcription factor interaction

To explore transcription factor and miRNA–mRNA interaction in particular modules, the database TargetScan Mouse (Release 7.1) (http://www.targetscan.org/vert_71/) was used for searching the predicted microRNA targets, and MicroCosm Mouse (Release 5) (http://www.ebi.ac.uk/enright-srv/microcosm/htdocs/targets/v5/) was used to build a miRNA–mRNA interaction network and the network was visualized with Cytoscape 3.3.8 software [46]. Putative miRNAs were generated by the intersection of the miRNA-targets from TargetScan and Microcosm. Transcription factors may control different gene modules. Therefore, we further used iRegulon Cytoscape plugin [47] to identify the possible transcription factors and their co-factors that are associated with a collection of intramedullary hub genes extracted from prognosticate modules (Supplementary Table 6, Supplementary Table 7, Supplementary Table 8, 9). To expand our network, we employed CyTargetLinker plugin [48]. CyTargetLinker is able to enhance biological networks using the provided information in frame of Regulatory Interaction Networks or RegIN. RegIN is a network in xgmml format containing regulatory interactions.

### Module preservation

To determine the reliability of the identified vascular aging-related modules and compare the modular structure in inferred co-expression network with a reference network generated based on an independent dataset, we used a previous study with GEO access number GSE10000 [17] to identify significantly differentially expressed genes (*P* < 0.05). In the dataset GSE10000, the two groups of samples corresponding to 32-week old wild-type mice (around 6-month old) (n=3) and 78-week old wild-type mice (around 20-month old) (n=3), were comparable with the first-stage samples corresponding to 6-month old mice (n=6) and 20-month old mice (n=6). Therefore, we removed the samples corresponding to 6-week old mice (n=3) and the ApoE-/- mice. Based on the significantly differentially expressed probe sets, we reconstructed a test co-expression network and contrasted the preservation of co-expression network across testing and reference datasets to detect the conservation of gene pairs between the two networks. Briefly, a Z-summary < 5 and lower median rank indicates weak preservation between the testing and reference networks.

### Animal breeding, mouse aorta isolation, and real-time polymerase chain reaction

All animal experiments were approved by the Animal Ethics Committee of Shanghai Jiao Tong University. Male mice (C57BL/6J) were obtained from the Shanghai Laboratory Animal Center, Chinese Academy of Sciences (SLAC, CAS), and kept for 7 days in the local animal house for acclimatization. The mice were 6 and 20 months of age old, housed at a constant ambient temperature under a 12h/12h light-dark cycle and supplied with distilled water, and pelleted AIN-76A (Research Diets, New Brunswick, NJ) chow ad libitum. Quantitative real-time polymerase chain reaction (qPCR) was used to validate the expression of genes changed in aortas from mice aged 6-month (n = 20) and 20-month (n = 20).

A mouse aorta was perfused in situ with cautious to keep the adventitia intact. The vessel was flushed thoroughly with ice-cold phosphate-buffered saline (PBS), through the left ventricle of the heart, cleaned periadventitial fat and connective tissues, snap-frozen in liquid nitrogen, and stored at −80 °C.

Total RNA, including miRNA, was extracted from the tissue using Qiagen RNeasy Mini Kit (Qiagen, Hilden, Germany). Total RNA from aorta was treated with DNase I to remove genomic DNA. RNA integrity was checked by Agilent2100 Bioanalyzer (Agilent Technologies, Palo Alto, CA) and RNA gel electrophoresis.

For the quantification of mRNAs, 1 μg of the total RNA was converted into cDNA using a reverse transcription kit (Promega) based on the manufacturer’s instruction. The gene expression of hub genes (Enpp5, Fez1, Kif1a, F3, and H2-Q7) were then detected by real-time qPCR. The qPCR were conducted using SYBR Premix Ex Taq (Takara, Japan) in 20 μl reaction solution containing 1μl cDNA, 10 μl SYBR Premix Ex Taq (2X), 0.4μl forward primer, 0.4 μl reverse primer, 0.4 μl ROX reference dye, and 7.8 μl ddH_2_O. The PCR amplification procedure was carried out on an ABI 7500 fast real-time PCR system (Applied Biosystems, USA) at 95°C for10 s, followed by 40 cycles of 95°C for 5 s and 60°C for 35 s. The amplification reaction without the template was used as a negative control. β-actin gene was used as the internal reference. Primers used for gene amplification are available on request.

The miRNAs (mmu-miR-449a, mmu-miR-449c, mmu-miR-34c, mmu-miR-34b-5p, mmu-miR-15a, and mmu-let-7) were detected by qPCR. Briefly, 2 μg RNA were performed using the miRcute miRNA First-Strand cDNA Synthesis Kit (Tiangen, Beijing, China). The qPCR was then conducted using the miRcute miRNA qPCR Detection Kit (SYBR) (Tiangen, Beijing, China) in 20 μl reaction solution containing 1 μl cDNA, 10 μl 2× miRcute miRNA premix, 0.4 μl 50× ROX Reference Dye, 0.4 μl forward primer, 0.4 μl reverse primer, and 7.8 μl ddH_2_O. The PCR amplification procedure was carried out on an ABI Prism 7500 Fast Sequence Detection System (ABI, Carlsbad, CA, USA) at 94°C for 2 min, followed by 40 cycles of 95°C for 20 s and 60°C for 35 s. Small nucleolar RNA (RNU6B) was used as the housekeeping loading reference. Forward primers were designed based on mature miRNA sequences while the reverse primers were provided by the miRcute miRNA qPCR Detection Kit (SYBR) (Tiangen, Beijing, China).

The relative gene expression was calculated using the 2^-ΔΔCt^ method [49] (Song et al., 2018). The experiments were conducted three times independently. Statistical comparison of the levels was analyzed using two-tail unpaired Student’s t-test, and differences were considered significant if *P* < 0.05.

## Supplementary Material

Supplementary Figures

Supplementary Table 1

Supplementary Table 2

Supplementary Table 3

Supplementary Table 4

Supplementary Table 5

Supplementary Table 6

Supplementary Table 7

Supplementary Table 8

Supplementary Table 9

Supplementary Table 10

Supplementary Table 11

Supplementary Table 12

Supplementary Table 13

Supplementary Table 14

Supplementary Table 15

Supplementary Table 16

Supplementary Table 17

Supplementary Table 18

Supplementary Table 19

Supplementary Table 20

Supplementary Table 21
