# Supplementary material for: Co-expression network analysis identified hub genes critical to triglyceride and free fatty acid metabolism as key regulators of age-related vascular dysfunction in mice
Source: Aging (Albany NY). 2019 Sep 12;11(18):7620–38. doi: 10.18632/aging.102275 (PMC6781998; doi:10.18632/aging.102275)
Supplement: Supplementary Figures [file aging-11-102275-s019.pdf]

SUPPLEMENTARY FIGURES

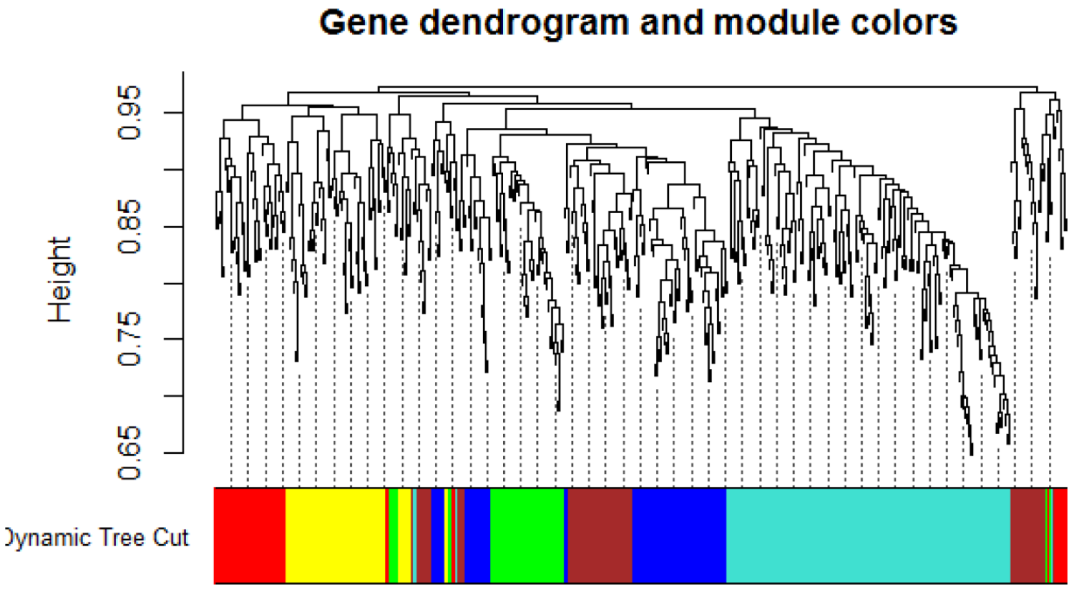

Supplementary Figure 1. Hierarchical clustering of genes in significant modules. The colors are assigned to each module by the Dynamic Tree Cut.

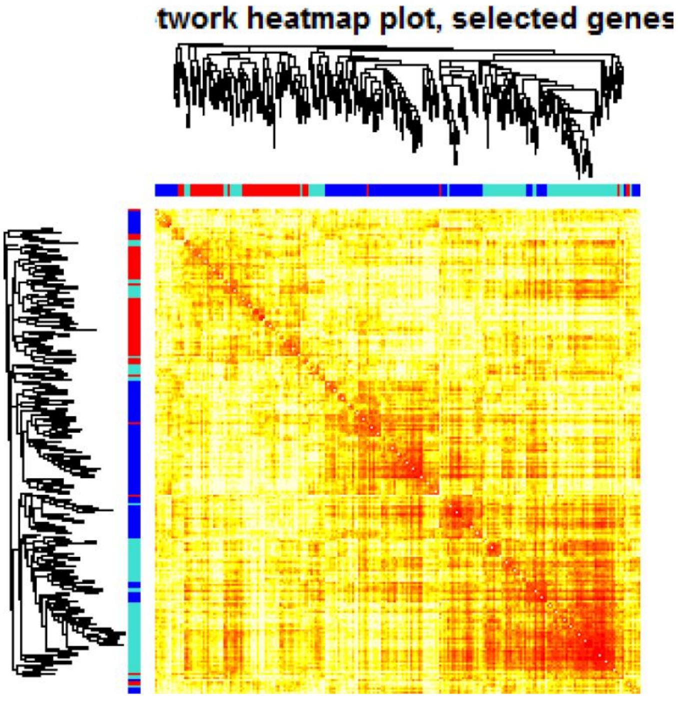

Supplementary Figure 2. Heatmap plot of topological overlap in the gene network. Each row and column corresponds to a gene, light color denotes low topological overlap, and progressively darker red denotes higher topological overlap. Darker squares along the diagonal correspond to modules. The gene dendrogram and module assignment are shown along the left and top.

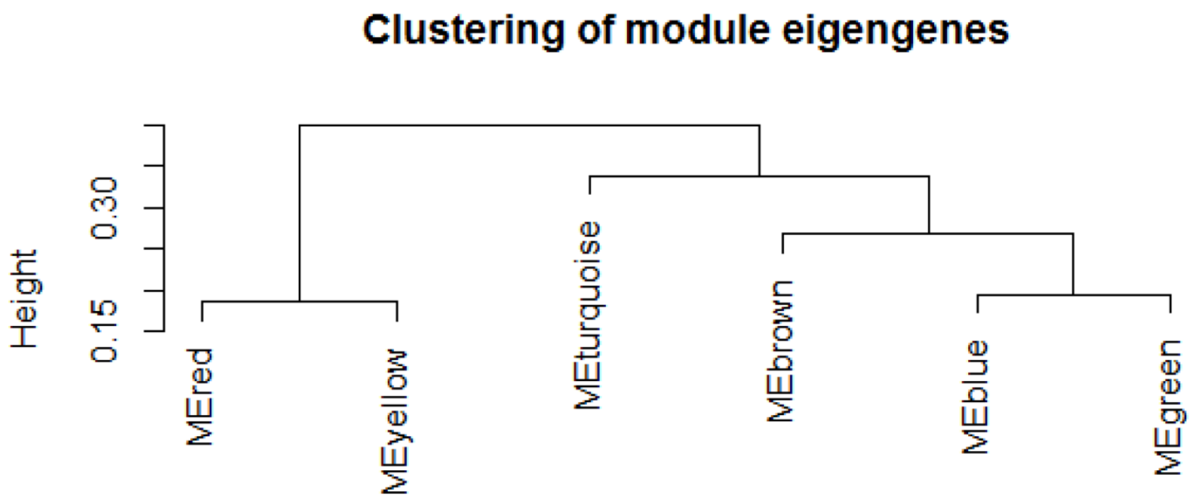

Supplementary Figure 3. Hierarchical clustering of module eigengenes that summarize the modules found in the clustering analysis. Branches of the dendrogram (the meta-modules) group together eigengenes that are positively correlated.

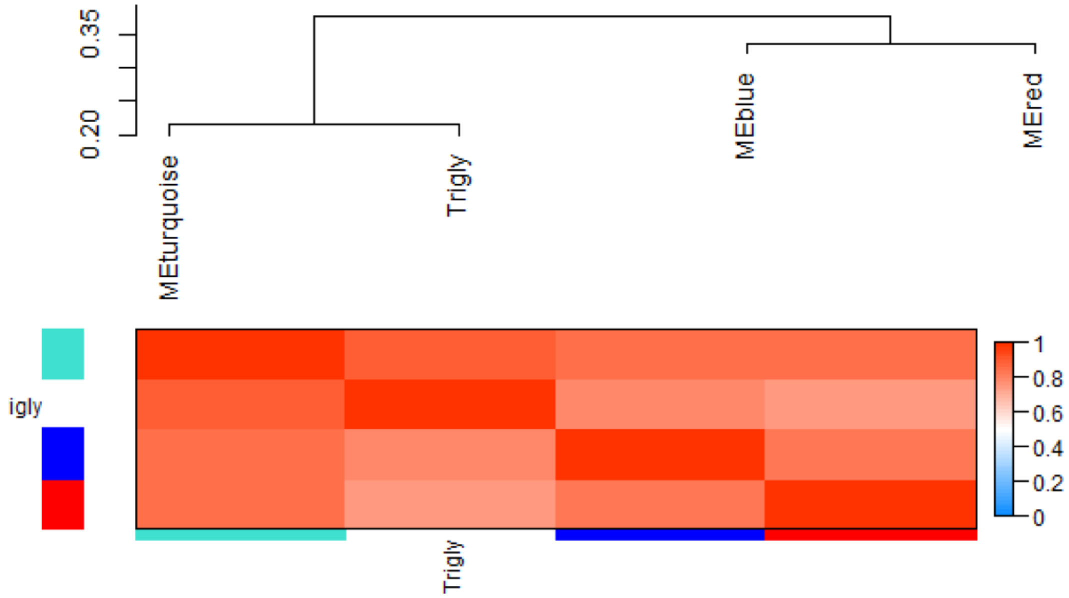

Supplementary Figure 4. Hierarchical clustering and heatmap of module eigengenes (labeled by their colors) and the triglyceride

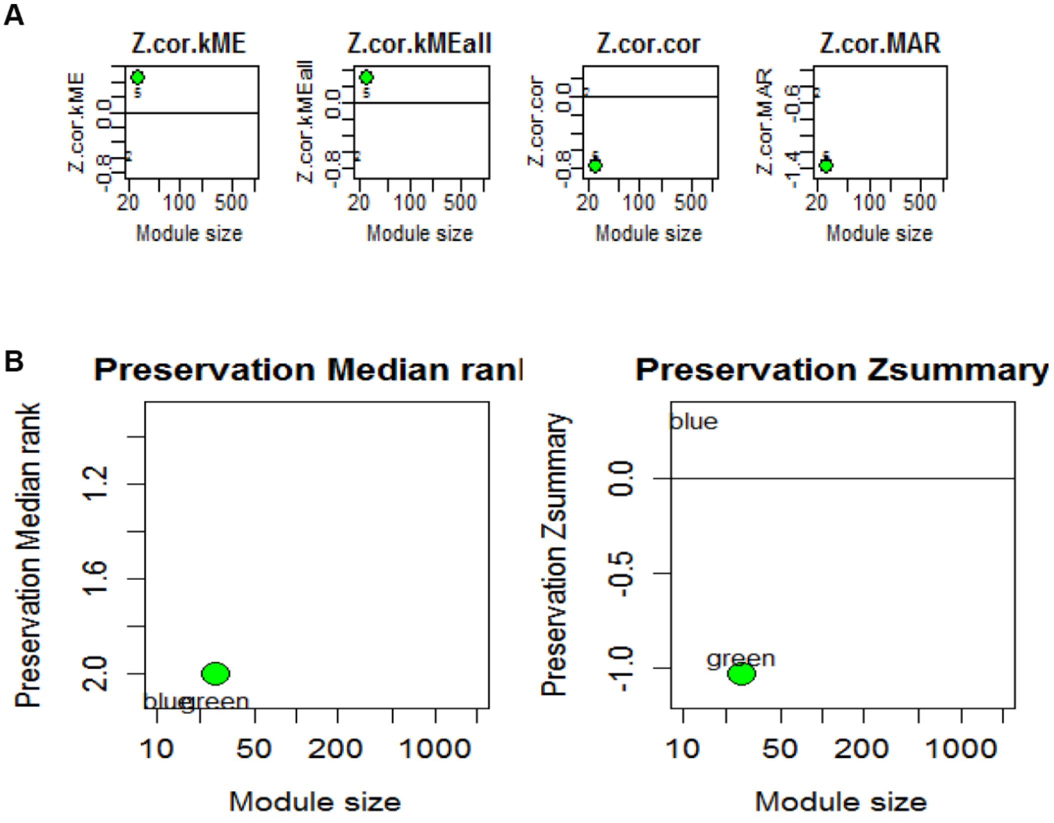

Supplementary Figure 5. Preservation statistics in age-related vascular aging data (GSE50833). The size of the bubble represents module size in the data subset used to assess module preservation. The horizontal lines indicate the Z-summary.preservation thresholds for strong evidence of conservation (above 10) and for low to moderate evidence of conservation (above 2). (**A**) Z-summary.preservation related to module size (**B**) relationship between the two preservation statistics (Z-summary.preservation and medianRank). Lower medianRank indicates higher preservation.

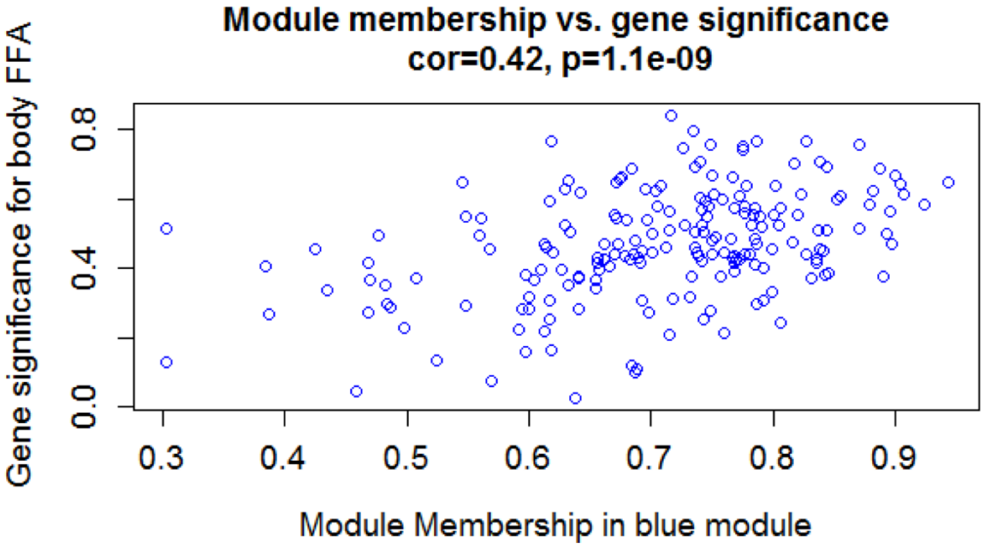

Supplementary Figure 6. Scatterplot showes a highly significant correlation between gene significant (GS) versus module membership (MM) in the blue module with free fatty acid (FFA).

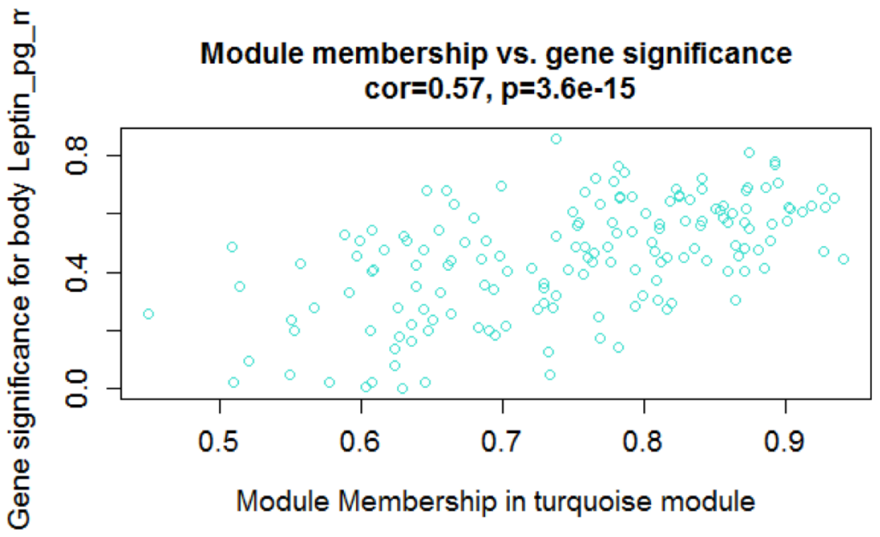

Supplementary Figure 7. Scatterplot showes a highly significant correlation between gene significant (GS) versus module membership (MM) in the turquoise module with leptin.

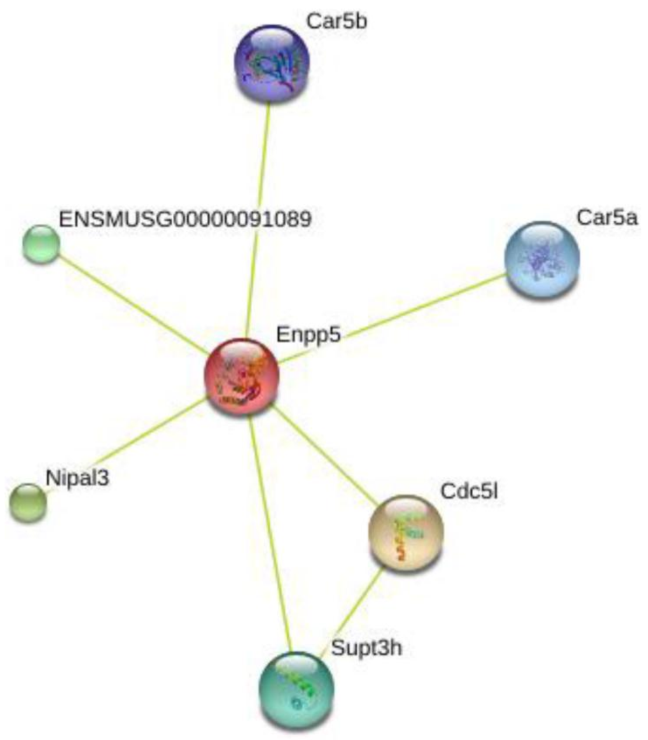

Supplementary Figure 8. Protein-protein interaction network of Enpp5 gene.
